# Usability evaluation of an eHealth intervention for family carers of individuals affected by psychosis: A mixed-method study

**DOI:** 10.1177/2055207619871148

**Published:** 2019-08-28

**Authors:** Jacqueline Sin, Luke A. Woodham, , Claire Henderson, Elen Williams, Aurora Sesé Hernández, Steve Gillard

**Affiliations:** 1 Population Health Research Institute, St George’s, University of London, UK; 2 School of Psychology & Clinical Language Sciences, University of Reading, UK; 3 Institute of Medical and Biomedical Education, St George’s, University of London, UK; 4 Health Service & Population Research Department, King’s College London, Institute of Psychiatry, Psychology & Neuroscience, UK; 5 Independent GP*Both of the authors are first joint authors on the published paper.

**Keywords:** Family, carers/caregivers, psychosis, eHealth, usability evaluation, think-aloud test, heuristic evaluation, System Usability Scale

## Abstract

**Background:**

Existing research suggests that eHealth interventions targeting family carers of individuals with long-term illness offer a promising approach to care delivery. In particular, digital psychoeducational interventions with interactive psychosocial support are well-received with high rates of satisfaction and acceptability. However, development of such interventions for psychosis carers is lacking. We developed a multi-component eHealth intervention specifically for carers of individuals affected by psychosis, called COPe-support (Carers fOr People with Psychosis e-support).

**Objective:**

Using mixed methods to evaluate usability, system heuristics and perceived acceptability, we conducted a usability study to establish the suitability of the intervention prototype for the target user group.

**Methods:**

Twenty-three carers were recruited to the study and participated in a think-aloud test or a remote online trial of the intervention. Qualitative feedback, post-use System Usability Scale (SUS) scores, and real-world usage data collected from the tests were analysed. These were also supplemented with heuristic evaluation data provided by an independent eLearning technology expert.

**Results:**

Participants evaluated the intervention content as useful and helpful, and indicated that the system had satisfactory usability with a mean SUS score of 73%, above the usability quality benchmark threshold. Study results identified some minor usability issues, which were corroborated with the eLearning expert’s heuristic evaluation findings. We used these results to refine the COPe-support intervention.

**Conclusions:**

The usability study with end-users and service providers identified real-life usage and usability issues. The study results helped us refine COPe-support and its delivery strategy before its launch as part of a large-scale clinical trial.

## Introduction

With ever-advancing healthcare technologies and growing longevity worldwide, a significant proportion of people provide substantial and sustained help and support to friends or family members suffering from a long-term illness.^1^ In the UK, it is estimated that nearly 25% of the general population identify themselves as a carer for a family member affected by a mental illness.^2^ This figure is similar to that reported in the United States, that up to 29% of adults are a carer for a relative who is ill, disabled or elderly.^3^ While informal caregiving (caregiving hereafter) provides paramount emotional and economic benefits to the cared-for individuals as much as to society as a whole, it is also well-established that caring demands can jeopardise the carers’ wellbeing.^4,5^

Large numbers of family and friends who provide care for a loved one with a long-term illness need and can benefit from information and support themselves.^6,7^ A recent systematic review on eHealth (or e-health) and mHealth (or m-health) interventions targeting carers supporting a loved one with a long-term illness identified a rapidly growing body of literature and suggested that resources delivered through the Internet can potentially address such needs.^8^ In addition to its popularity, the existing research evidence further indicates that carers largely perceive eHealth intervention as accessible, desirable and helpful. The most common approach identified comprises psychoeducation intervention delivered via an enriched online environment with supplementary modes of communication, such as online support with healthcare professionals and peers. As many carers are in a busy phase of their life, working and possibly raising a family of their own while fulfilling their caregiving role, they particularly appreciate the flexibility and self-paced nature of information about the illness condition and the support for them delivered through eHealth interventions at a low cost.^8–10^ Indeed such eHealth interventions may provide a lifeline for carers, who in most healthcare systems are not entitled to healthcare support in their own right.^3,11^

### Carers supporting a loved one with psychosis

Psychosis, or psychotic disorders such as schizophrenia, are the most common and severe mental illness, with a lifetime morbidity risk (that is, the number of people estimated to develop a psychotic disorder at some point in their life) estimated to be approximately 1%.^12,13^ In the UK alone, it is estimated that approximately a quarter of a million individuals are suffering from psychosis at any one time.^14^ Psychotic symptoms are often distressing and frightening; these include hallucinations: false sensations like hearing voices or seeing things that do not exist; delusions: false beliefs such as one is being conspired against or being persecuted by an external force; and secondary and/or co-morbid mood and anxiety problems.^12^ As the onset of psychosis often peaks around late teenage years affecting the individual’s mental, social and occupational functioning, and the treatment is commonly required over a long period, it is widely recognised that coping with a psychotic illness can be challenging and difficult not just for the individual but for everyone closely related to them.^5,6,15^

The significance of family members and friends in mental healthcare (commonly referred to as carers) is also well-established, in that individuals in receipt of support and care from their social network have a better prognosis and an enhanced quality of life.^16–18^ On the other hand, carers themselves are often found to have higher vulnerability and morbidity of mental and/or physical ill-health, in part due to the burden of caregiving.^2,17^ Indeed, distress in carers frequently reaches clinical thresholds, and their psychological morbidity scores (such as depression and anxiety) are found to be consistently associated with the amount of care they provide; that is, as the amount of care increases, the health of the carers worsens.^2,19^ Furthermore, extant research evidence also suggests that carers’ wellbeing or rather lack of it could hamper their caregiving capacity. Carers who feel they are not supported and lack the resources to cope are less likely to engage in caring for their loved ones, or more likely to exhibit critical or hostile behaviour towards the cared-for, albeit unintentionally, which in turn jeopardises the patients’ recovery.^4,5^ There are clear and pressing needs for effective interventions targeting carers.

### Psychoeducational eHealth interventions

Enhanced psychoeducation, that is, information-giving on the illness condition and related caregiving and problem-solving strategies (delivered via a face-to-face medium) targeting carers of individuals affected by psychosis, has a strong evidence-base for its effectiveness in enhancing carers’ knowledge and coping with their caring roles.^6–8^ In addition to information and advice given by mental health professionals, carers also identified peer-to-peer support as particularly useful in reducing their sense of isolation.^7–9^ Indeed, carers have expressed their need for such support to be delivered to them through a digital medium for optimal flexibility and accessibility, so as to fit in with their many other commitments in addition to caregiving.^8,11,20,21^

Over the last decade, in line with the increasing popularity and availability of eHealth (i.e. healthcare practice delivered via the Internet) and mHealth (i.e. via the mobile network) technology, advances have been particularly notable in the field of dementia and eating disorder caregiving.^8,11,20^ Carers appreciated the flexibility, self-paced nature and individualised programme of information and support of such interventions. They also highly rated the network support function, which is a common feature integrated in many interventions through an online carer forum and/or a space to consult healthcare professionals. Furthermore, eHealth interventions are perceived to be more advantageous than their counterparts delivered via a face-to-face medium arguably because they often place a stronger emphasis on users’ inputs in terms of product content and usability. As eHealth interventions are designed to be used autonomously by users in their own natural content ultimately,^21,22^ it is therefore essential to establish their accessibility, usability and likability. As such, usability studies and iterative consultations with end-users are unique study designs used along the intervention-development pathway.

In contrast, much less progress has been made for carers supporting a loved one with psychosis, with only a few empirical studies documented to date.^22–26^These studies used either a pilot trial or a usability study design to test a digital resource providing psychoeducation with or without moderated peer discussion, with a relative small sample of carers. Although the results from the aforementioned studies were favourable, there have been no further definitive studies on any eHealth interventions targeting psychosis carers to date. This demonstrates the relative infancy of the evidence base of eHealth intervention effectiveness in the field currently.

**Figure 1.**
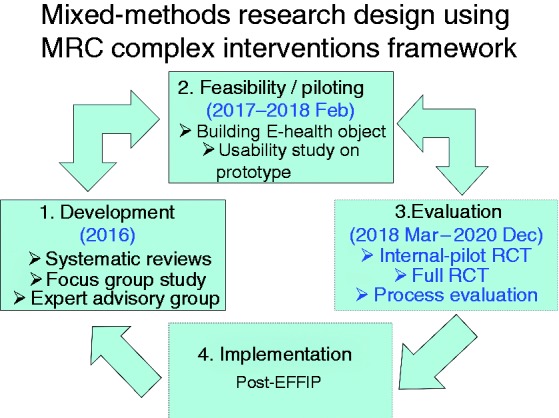
Mixed-methods research design using MRC complex interventions framework.

Hence, the EFFIP (E-support for Families and Friends of Individuals affected by Psychosis) Project was initiated to fill these research gaps by developing and evaluating an Internet-delivered, multi-component eHealth intervention for carers supporting a loved one with psychosis.^27^ We designed the intervention using mixed-method studies staged within the Medical Research Council (MRC) Complex Interventions Framework.^28^ The MRC framework defines health interventions as those comprising a number of, and interactions between components within the intervention, those components subsequently impacting on a number of outcomes spanning behavioural, cognitive and emotional domains.^28^ We applied the framework in three phases, integrating theoretical and empirical research work to inform the design, development and modelling of the intervention (see Figure 1). The first phase – theoretical development of the digital intervention – was informed by empirical studies including a focus group study and reviews of existing research data. The building and usability evaluation of the intervention lay in the second phase, that is, feasibility and piloting. Lastly, the clinical effectiveness of the intervention on the carers’ wellbeing and other health outcomes is to be investigated through a randomised controlled trial, in the evaluation phase.

This paper focuses on reporting the usability evaluation of the intervention prototype. We considered various established usability study methods for testing digital health intervention usability and decided to employ a mixed-method approach to collect comprehensive data on views and feedback from the end-users (i.e. carers) and an expert in eLearning.^29–31^ The study aimed to explore multiple perspectives from both end-users and experts in the field on the usability, feasibility and acceptability of the intervention prototype. Specific objectives included:

- To identify carers’ (as end-users) feedback on the usability, feasibility and acceptability of the intervention prototype;
- To collect usage data to better understand real-life usage patterns of the intervention;
- To collect expert opinion on the heuristic usability (i.e. software design and navigation) of the prototype; and
- To identify strengths and weaknesses of the prototype, and areas in need of further development and refinement in finalising the intervention.

## Methods

The usability evaluation study included three sub-studies each using a unique method, as follows:

1. Think-aloud test;
2. Remote usability test; and
3. Heuristic evaluation by an expert.

The use of a mixed-methods design allowed us to address different dimensions of usability, and to identify specific areas for iterative improvement from the perspective of both target end-users and usability experts. Each method is able to yield insights around different aspects of usability that may ultimately impact upon the effectiveness of the intervention, and can complement each other to allow us to identify both potential usability issues and propose solutions to these. This is not possible when using one approach alone.

The study was approved by the UK National Health Service Research Ethics Committee process (REC approval reference number: 17/LO/1642) and Health Research Authority (HRA IRAS project ID: 233238). Carer-participants were recruited from three NHS mental health trusts based in South East England. All participants gave their consent prior to joining the study.

### The intervention prototype

The digital health intervention prototype under usability evaluation was developed through the EFFIP project.^27^ The intervention is delivered through a web-based virtual learning environment (VLE) called Canvas (https://www.canvasvle.co.uk/). The Canvas VLE, and thus the COPe-support intervention, is designed to be accessible via desktop or laptop web browsers, as well as smartphones or tablets through a Canvas app.

The development of COPe-support was informed by the results from the earlier studies conducted in the theoretical development and modelling/feasibility phases of the overall EFFIP project.^27^ The theoretical development phase comprised two stages. First, we conducted two systematic reviews. One systematic review investigated the effectiveness of psychoeducational interventions that used any type of delivery method on carers’ wellbeing and health morbidities, and also looked at the correlation between intervention duration, dosage and effectiveness.^6^ The second review focused on scoping eHealth interventions targeting family carers of people with long-term illness.^8^ Through this review, we examined the common information and communication technology (ICT) features and implementation considerations used in such interventions. Secondly, we conducted a focus group study with individuals affected by psychosis and family carers to explore their views and ideas for the optimal intervention design. We then meta-synthesised findings from these studies to inform the design and content of our intervention.^29^

Theoretically, COPe-support is based upon the stress-appraisal and coping theory^30^ that is commonly used in conventional psychoeducational interventions targeting family members and relatives (i.e. face-to-face delivery). COPe-support works by imparting information about psychosis and related caring strategies as well as providing support shared between carers as peers, so as to enable them to be more self-efficacious in coping with the caregiving demands and hence, achieve better wellbeing.^6,16^ Further digital health heuristic considerations and eHealth intervention behaviour-change techniques were identified and integrated into the design of COPe-support. These included digital automated functions and ICT communicative functions including the use of an enriched information environment with inbuilt discussion forums with peers and professionals.^31–34^

The content of COPe-support is grouped into 12 sections: a Home page with introduction and navigation guidance; eight sections organised by topics focusing on information-giving on psychosis and related caring issues (readable as HTML documents or downloadable as fillable pdf documents); two online forums: one called ‘Ask the Experts’ where carers can post questions for advice from a panel of healthcare professionals or experts by experience (e.g. individuals with lived experience of living with psychosis); and the other called ‘Peer to Peer’ where carers exchange views with peers; and a ‘Further Resources’ section. Throughout the intervention, there are cognitive-behavioural orientated exercises and reflection points designed to encourage participants to take stock of wellbeing-promotion and caregiving skills and to integrate these into their own life. The content contains a mixture of textual and audio-visual information devised by the study team with contributions from experts by experience (both ex- and current service users who have lived experience of psychosis and carers) and clinical-academic experts.

**Figure 2.**
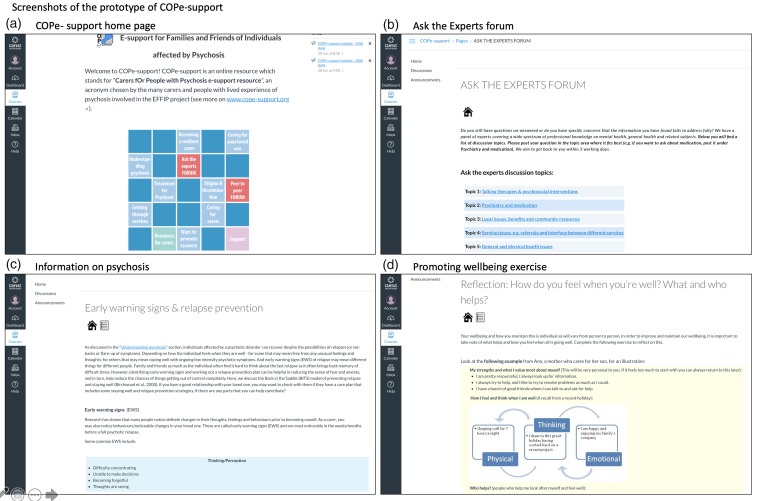
Screenshots of the prototype of COPe-support.

To create and maintain a secure and safe online environment, participants were required to follow a set of ground rules in using COPe-support. First, while participants needed to provide their personal information as part of the eligibility screening, consent and enrolment process, we asked them to choose a pseudonym and use it on COPe-support for anonymous participation. Second, we asked all participants to observe the confidentiality principles in sharing their own and their cared-for individual’s details when using the intervention. We provided guidelines to explain what constitute personal (or person-identifiable) data and how participants can fully participate in the interactive forums (e.g. writing a post about their story or raising a question) without giving any such data away, to preserve their and their family’s anonymity. Third, all participants had to respect one another in their communications. Last but not least, an online facilitator (JS), a mental health nurse with over 20 years’ experience specialising in psychosocial interventions for people with psychosis and their family carers, monitored and moderated the online intervention daily during the week. JS also posted weekly updates via the COPe-support announcement function to all participants with an aim to keep them engaged (the system also populated an email of the update, which was sent to their email address). The intervention can be accessed through computer (desktop or laptop) as well as mobile devices (e.g. tablets and mobile phones). For the usability study, we released the prototype with 70% of the intended final content active and live (see Figure 2 for sample screenshots of the COPe-support prototype).

## Participants

For the think-aloud test and the remote usability study, we recruited carers who were supporting a loved one affected by psychosis (i.e. end-users of COPe-support) and residing in South East England. Eligibility criteria were: age 18 or over; have at least weekly contact in any format with their cared-for individual; able to use usual Internet communications in English; and have regular Internet access. Potentially suitable and interested participants were screened for eligibility before informed consent was obtained online. All carer-participants were given a £10 goodwill payment to compensate for their time and contributions. We invited three carers for the think-aloud test, which was the first study to be conducted in November 2017. We then recruited 20 carers to join the remote usability test during January 2018. The sample sizes for the think-aloud test and the remote usability study were established according to the principles set out by experts in the field and prior usability studies using similar methods.^35,36^ For the heuristic evaluation of the prototype, one eLearning expert (LW) conducted the assessment in December 2017.

## Data collection procedures

### Think-aloud test

Think-aloud tests (or test sessions) are the single most frequently used method to evaluate the usability of digital systems.^31,33,37^ Researchers use think-aloud tests to expose the users to the digital system while asking them to verbalise their thinking so as to get “users' inferences, intuitions, and mental models, reasons, decisions, … while doing the tasks”.^37^ In this study, we conducted the think-aloud test with three carers on an individual basis on the intervention prototype. We invited the carers to log into an online version of the COPe-support prototype using a desktop computer with a designated account created for their use at the study team base (South London). During a test-session lasting about an hour each, the participants were asked to browse through COPe-support and try using all the available functions. All actions performed by the participants (e.g. pages visited and posts made in the forums) were recorded automatically and non-intrusively into the Canvas system. The think-aloud session was also audio-recorded to capture the users’ experience whilst using the prototype. We analysed the transcribed audio-recorded inferences using content analysis method^38^ to understand participants’ general perception of the prototype and to identify key areas of strengths and weaknesses. In brief, the transcripts were initially read through once without being coded. Transcripts were then read and reread systematically to identify codes and then themes at a higher level. Two authors undertook the analysis to optimise the accuracy of the analysis.^38^

### Remote usability test

Twenty carer-participants consented and completed the online enrolment procedure that was available on the project website between November 2017 and early January 2018 to join the remote usability test. We arranged a login for the participants, which they could activate with a self-chosen password and then use to access all the available content and functions of the intervention over a two-week period in late January 2018. Participants had 24/7 remote access to the COPe-support VLE platform using their login from their own home. We recommended that participants spent about an hour per week over the study period using the intervention, picking the content most relevant to their own needs and circumstances. The study facilitator spent about an hour a day monitoring and moderating the two interactive forums. The participants’ usage of COPe-support, such as number of logins, date and time of logins, pages visited and posts made on the two forums, were automatically recorded by the Canvas system usage statistics. At the end of the study period, participants were invited to complete an evaluation questionnaire online.

### Outcome measures

After the carer-participants had given their consent to join either study, they were asked to provide demographic data (including their age, gender, and caring situation such as living with a cared-for individual or not) via an online enrolment process.

To collect carers’ views on the usability and acceptability of the COPe-support prototype, we devised an evaluation questionnaire for their completion immediately after using the prototype. The questionnaire was adapted from an earlier study testing an eHealth psychoeducational intervention.^24^ It comprised questions eliciting participants’ perceptions of the usefulness, helpfulness and acceptability of the intervention, through their rating on a 5-point Likert scale (e.g. very helpful, quite helpful, neither, quite unhelpful, very unhelpful). There were also free text entry spaces for the participants to give reasons behind the rating they gave and for general feedback. Furthermore, we incorporated the System Usability Scale (SUS),^39^ a ten-question web application usability scale, within the evaluation questionnaire. The SUS contains a mix of positive and negative items pertaining to different usability aspects of web applications, including effectiveness (i.e. the ability of users to complete tasks using the system), efficiency (i.e. the level of resource consumed in performing tasks), and satisfaction.^39^ For each question, the participant rates the magnitude of their agreement using a 5-point Likert scale with statements ranging from strongly disagree (1) to strongly agree (5). Total SUS scores range between 0 and 100. Higher values reflect higher user satisfaction and interventions scoring 68 or above are regarded as above-average in terms of usability quality.^39,40^

### Heuristic evaluation

In order to identify the most significant usability problems from the perspective of accepted principles of good usability design, and to triangulate these findings with those from the other user-centred approaches to usability evaluations, one eLearning expert (LW who remained independent from the build work of COPe-support) conducted an evaluation using the 10 heuristics developed by Jakob Nielsen.^41,42^ Nielsen’s heuristics are widely recognised as the general benchmark for good interface design.^29,42^ These 10 heuristics include: visibility of system status; match between system and the real world; user control and freedom; consistency and standards; error prevention; recognition rather than recall; flexibility and efficiency of use; aesthetic and minimalist design; help users recognise, diagnose and recover from errors; and help and documentation. We further adapted the usability issue severity ratings commonly used in such heuristic evaluations, which normally involve multiple usability experts. Our overall impact rating combined a three-level frequency rating for measuring frequency of appearance of a usability problem (1 = only in one place, 2 = in several places, and 3 = as part of the main persistent navigation interface) with Nielsen’s 5-point ratings for severity (from 0 = no usability problem at all, to 4 = usability catastrophe).^41,43^ This means the overall impact scores ranged from 0 to 12; the higher the score, the more severe and widespread a heuristic is.

## Results

In total, 20 carers participated in the study; three undertook both the think-aloud test and the remote usability test and the remainder the remote test only. Just over half of the carers (*n* = 11, 55%) lived with their cared-for person. Most of the participants were a parent (*n* = 13, 65%), and there were five spouses (25%), one sibling, and one adult-child. The age range of participants was 27–80 years (mean = 56.4, SD = 9.9). Half of the participants were in full-time (*n* = 7) or part-time work (*n* = 3); the rest were either retired (*n* = 7) or not engaged in gainful employment (*n* = 3, including being a full-time carer) at the time of the study. The participants comprised 8 men and 12 women. The gender mix of the cared-for persons was similar to that of the participants; 9 were male (45%) and 11 were female. The ages of the cared-for persons ranged from 19–63 years (mean = 35.6, SD = 9.9). Half of the cared-for persons (*n* = 10, 50%) had a diagnosis of psychosis, seven were diagnosed with a schizophreniform disorder, and three type 1 bipolar disorder. As reported by the carer-participants, the cared-for persons had been unwell for less than one year to 30 years (median = 8 years). Eight of the 20 cared-for persons had developed psychosis within the previous three years, meeting the criteria of first episode psychosis. This means that 40% of the carer-participants were relatively new to adopting a caring role. The demographic characteristics and caring situation of the participants and their cared-for persons are summarised in Table 1.

**Table 1.** Summary of study participants’ demographic characteristics and caring situation.

Characteristics	Carer-participants (*n* = 20)	Their cared-for person (*n* = 20)
Age: mean (SD)	56.4 (9.9)	35.6 (9.9)
median (range)	56.5 (27 – 80)	36.5 (19 – 63)
Sex: male (*n*/%)	8 (40%)	9 (45%)
Ethnicity (*n*/%)		
White	14 (70%)	
Mixed	2 (10%)	
Black	3 (15%)	
Other	1 (5%)	
Work (*n*/%)		
Full-time work	7 (35%)	
Part-time work	3 (15%)	
Retired	7 (35%)	
Not working	2 (10%)	
Looking after home/family	1 (5%)	
Education (*n*/%)		
Trade training	8 (40%)	
Degree and post graduate	11(55%)	
Other professional qualification	1 (5%)	
Marital status (*n*/%)		
Single	6 (30%)	
Married/cohabiting	12 (60%)	
Other	2 (10%)	
Relationship with the cared-for person (*n*/%)		
Parent	13 (65%)	
Spouse/partner	5 (25%)	
Sibling	1 (5%)	
Child	1 (5%)	
Accommodation arrangement of carer (*n*/%)		
Live with cared-for person	11 (55%)	
Not live with cared-for person	9 (45%)	
Diagnosis of cared-for person (*n*/%)		
Psychosis		10 (50%)
Schizophreniform disorders		7 (35%)
Type 1 bipolar disorder		3 (15%)

### Think-aloud test results

Three carers (two women and one man) participated in the think-aloud test individually with one or two of the study team members (JS or AS). The test sessions lasted between 34 and 44 min (mean = 38, SD = 5.6), and the participants managed to swiftly complete all the tasks listed below:

- To activate a designated account on Canvas to access COPe-support;
- To find relevant information about psychosis and its treatment on COPe-support;
- To post a question on the ‘Ask the Experts’ forum, following the ground rules and guidance;
- To post a discussion point on the ‘Peer to Peer’ forum, following the ground rules and guidance;
- To download a reflection exercise and complete it as an interactive pdf; and
- To log off the prototype platform.

However, two out of three participants struggled with one task to ‘request support from/initiate contact to the online facilitator’ as they could not find the functionality to do so.

The qualitative data collected from the test procedure yielded individual codes that were combined into three broader themes. These are illustrated below with pseudonymised quotations.

### Intervention goals matching carers’ needs

All three participants were positive about the intervention comprising information-giving on psychosis and related caregiving strategies. They identified that such a provision would meet carers’ needs, as described by two participants:

This definitely gives me more information than I had when we were doing it a year ago. Finding information on psychosis was really, really difficult … This is so much more useful than anything we found on the web when we were looking (User 102).

This is something that I would have definitely found useful (User 101).

Participants also appreciated that the intervention focused on the carers themselves, offering them peer support and guidance on wellbeing-promoting strategies. Carers highly valued the mutual understanding and sharing among themselves through the interactive forums.

I think that’s what we need because we can go to forums for carers but having it online as well in your own home where you can just talk to someone about an issue, brilliant, fantastic. It makes me quite emotional actually, is the fact that for me it makes me feel like it’s not just my son, you know? (User 102).

### Navigation and usability

While going through the COPe-support site and performing the tasks, the participants rated the navigability of the online intervention positively and identified it as being easy and straightforward to use. In particular, participants appreciated the site structure and its main menu using a graphic design that was perceived as inviting and user-friendly (see Figure 2a).

It looks very clear to me. I like the structure (User 101).

Nonetheless, some navigational issues concerning finding their way (back) to the Home page were identified by the participants, together with some suggestions for improvement:

Sometimes they do have like ‘home’ written on there, don’t they? But I’m not sure it’s clear. I think I’d probably be looking for the word home at that stage (User 102).

Another functionality issue concerned the lack of clarity about ways to contact the online facilitator (for support) and to make a post on either forum, as Canvas had an inbuilt generic function for each that we could not change to suit our intervention design. In the think-aloud test sessions, participants could not easily find the functionality on Canvas, or struggled to master it:

I think I probably think that help is for when I can’t work the computer … I don’t think I’d go to that help button for emotional support or contact someone (User 102 on the task to send a message to the online facilitator).

What I didn’t notice is how to write a post, you see, … because here it should be like it would be a new thread wouldn’t it [not ‘Write a reply’ as it is shown on the screen] (User 101 on the task of making a post on the forum).

### Site environment

Overall, the participants perceived the site environment as safe, facilitative and inviting for carers to use. Their evaluation was based on the site environment with the ground rules and moderation integral within it:

Yes, I think that’s good. I think you’ve got to explain that it’s [COPe-support] not 24/7 and what is available 24/7 which we can see on the site (User 102).

And I think that [the ground rules and moderation] really helps as I can share and get some support from others without feeling too exposed because … otherwise I’d feel uncomfortable and worry that exposes my son as well, so I think that’s really good (User 103).

## Remote test results

### Remote test usage metrics

Out of the 20 participants, 16 activated their unique login and accessed the prototype during the study period. Collectively, they made 102 discrete logins to the COPe-support VLE platform (mean = 6.38, SD = 4.1, median = 5) over the two weeks. The number of logins by any one participant ranged from 2 to 13 times. Most participants visited multiple sections of COPe-support at each visit (or episode of login), thus generating a cumulative page-views figure of 2659 across the prototype over the whole study period. Page-views made by individual participants ranged from 2 to 462 and averaged at 166 per participant (SD = 154, median = 113).

Over the study period, a total of 45 posts were made on the two interactive forums, including 15 by the study facilitator and/or expert panel members in response to participants’ questions. Eleven participants made at least one post and the usage data showed all participants visited the forums repeatedly over the study period to read the updates. Table 2 summarises the number of posts made under the various topics of the two forums.

**Table 2.** Summary of number of posts made on COPe-support forums.

Ask the Experts forum		Peer to Peer forum	
Topic	No. of posts	Topic	No. of posts
Talking therapies	10	Coping with difficult emotions	0
Psychiatry and medication	4	Stigma	6
Legal issues	3	‘Looking after myself’	0
Service interface	2	Family dynamics	8
General health issues	4	Adjustment to loss and grief	0
Stigma and campaigning	0	‘The silver lining’: the good things out of it	12

### Remote test participants’ feedback

Fourteen participants (out of the 16 who had activated their login to COPe-support prototype) completed the online evaluation questionnaire within one week of completing the remote usability test. The responses are summarised in Table 3. With regard to intervention content and its relevancy and helpfulness, all the participants (*n* = 14, 100% for relevancy; *n* = 13, 93% for helpfulness) evaluated the intervention highly. The ease of use was also rated positively by the majority of participants (*n* = 12, 86%), with one each finding the system neither easy nor difficult, and difficult to use respectively. Participants were also asked specifically for their feedback on the ground rules and confidentiality measures incorporated in COPe-support; 79% of participants reported that they felt comfortable and secure in using the intervention, 64% found the ground rules helpful in maintaining a safe VLE environment.

**Table 3.** Evaluation questionnaire on COPe-support prototype usefulness, helpfulness and acceptability.

Evaluation items	Rating: See each individual item				
	*N* (%)	*N* (%)	*N* (%)	*N* (%)	*N* (%)
**How helpful was it for you to use the resource?**	Very helpful	***Quite helpful***	Neither	Quite unhelpful	Very unhelpful
	6 (43%)	***7 (50%)***	1 (7%)	0 (0%)	0 (0%)
**How relevant did you find the resource content to you in your caring role?**	***Very relevant***	***Quite relevant***	*Neither*	Quite irrelevant	Very irrelevant
	***7 (50%)***	***7 (50%)***	*0 (0%)*	0 (0%)	0 (0%)
**How easy was it for you to use the resource?**	***Very easy***	***Quite easy***	Neither	Quite difficult	Very difficult
	***6 (43%)***	***6 (43%)***	1 (7%)	1 (7%)	0 (0%)
**How helpful did you find the ground rules and guidance provided by the resource?**	***Very helpful***	Quite helpful	Unsure	Quite unhelpful	Very unhelpful
	***6 (43%)***	3 (21%)	*5 (36%)*	0 (0%)	0 (0%)
**How comfortable were you in sharing information about your situation on the resource platform in relation to its mechanism to maintain confidentiality?**	***Very comfortable***	Quite comfortable	Neither	Quite uncomfortable	Very uncomfortable
	***7 (50%)***	4 (29%)	3 (21%)	0 (0%)	0 (0%)
**Would you recommend this resource to other others?**	***Definitely***	Probably	Unsure	Probably not	Definitely not
	***10 (72%)***	3 (21%)	1 (7%)	0 (0%)	0 (0%)

Note: The mode response to each evaluation item is highlighted in bold and italic text.

Participants’ overall evaluation of COPe-support was positive: all bar one (*n* = 13, 93%) of the participants would ‘definitely’ or ‘probably’ recommend it to other carers. In particular, participants valued its web-based design and delivery, which meant that they could access and tailor the support and information package to suit their own needs and schedules. Participants commented that the intervention helped to engender an online community and resource for carers who often feel isolated yet busy with multiple commitments. Participants’ qualitative comments alongside their overall evaluation of the intervention included:

The info[rmation] provided is very helpful and the sections are well titled so you know what should be contained within them. The mechanics could of course be a little smoother perhaps, but this didn’t bother me too much (User 113, a mother).

I think the biggest help is talking to others in similar situations (User 117, a mother).

[It] helps to have a strong community of carers to communicate very easy[ily] among each other and able to seek help and advice (User 112, a spouse).

It contains lots of useful information and the contributions from carers provide authenticity to the site (User 120, a father).

I had a few difficulties getting to the login as I was using my smartphone but once logged in all was very easy (User 114, a mother).

## SUS analysis

**Figure 3.**
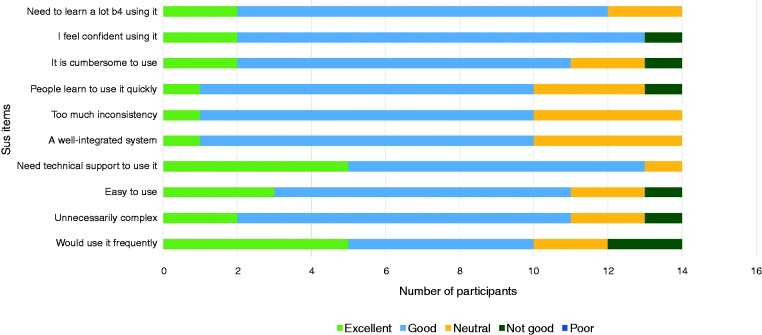
Summary chart of SUS item results.

A diagrammatic summary of participants’ usability evaluation of the intervention prototype across the 10 SUS items is presented in Figure 3. We then computed the SUS scores for all participants who completed the online questionnaire after trialling COPe-support (*n* = 14). The individual SUS total score ranged from 43% to 95%. The mean usability score based on the subjective evaluation was 73% (SD 13%). Twelve (out of 14) participants scored above 68%, the widely recognised usability quality benchmark.

### Heuristic evaluation results

The overall impact ratings for the 10 heuristics, with problems highlighted, are summarised in Table 4. Overall, no severe usability issues were identified within the COPe-support system. Therefore, the system was considered fit for purpose and able to be used for the purpose it was intended.

**Table 4.** Summary of heuristic evaluation results.

Heuristics	Problem description and comment for rating	Frequency	Severity	Overall impact
1. Visibility of system status	No usability issues that violate this heuristic have been noted.	0	0	0
2. Match between system and the real world	In both forums, the box to post message is labelled ‘Write a reply’ no matter if there was nothing to reply to or if the participant wanted to start a new post.	2	3	6
	In the Ask the Experts forum, ‘Notes for participants’ are provided. However, it is unclear whether the users will identify themselves as participants as, elsewhere in the site, users are addressed as ‘you’.	1	3	3
3. User control and freedom	There are no Home and Section index buttons on discussion pages. Without these, navigation is unclear, in ways to go back to the main course structure.	2	3	6
	When opening the introduction video, it opens on a new site in a new tab without the user necessarily realising that.	1	2	2
4. Consistency and standards	The Home and Section index icons use gradient buttons, which are not used elsewhere in the system. They do not change colour on hover-over, unlike other buttons in the system.	3	2	6
	The use of Section is not necessarily a widespread term in the system (as Canvas calls it a ‘module’, but it is consistently used in the site.	2	1	2
	Some text is underlined for emphasis, which inadvertently makes it look like a weblink.	1	2	2
	The end of the section page does not provide Home and Section index pages, unlike all other pages, instead giving a text link to the Home page while no link to section index page at all.	2	2	4
5. Error prevention	When a user tried to access a page not permitted by the site, the system handled this using well-described messages.	0	0	0
6. Recognition rather than recall	Introductory guides to navigation are available on the site; navigation elements are visible at all times.	0	0	0
7. Flexibility and efficiency of use	The site provides multiple routes of navigation that can be used depending upon user preference and style of use.	0	0	0
8. Aesthetic and minimalist design	The site is attractive and designed in a consistent way. There are standard Canvas features and functionality shown though not used by COPe-support.	2	2	4
	On physical health page, the text states ‘Watch the video’ unnecessarily as it is clear that the video is to be watched.	1	1	1
9. Help users recognise, diagnose and recover from errors	Error messages show very rarely if ever and are generally clear.	0	0	0
10. Help and documentation	The system is designed to be used without regular reference to documentation. Guidance is provided in the form of an introductory navigation video.	0	0	0

The heuristic evaluation results identified several minor usability issues, such as inconsistent use of terms (e.g. the inter-changeable use of participants, users or you) and the inconsistent appearances of ‘Home’ and ‘Section’ index buttons. There were also issues relating to the innate features of the Canvas platform, which was originally designed for eLearning courses. These included the standardised icons showing ‘Course stream’ and ‘Recent feedback’ on the right-hand side of the Home page display, and the standardised button entitled ‘Write a reply’ to initiate a post in the forums. The structured nature of the heuristics used to evaluate the tool meant that, by applying them to the platform to identify usability issues compared to best practice guidelines, the heuristics clearly implied solutions to these issues, which we were able to implement. We compared and combined the findings from the heuristic evaluation with the feedback from the think-aloud and remote usability tests and used these to inform the modifications required in order to resolve the identified usability challenges.

## Summary of modifications

We addressed all the issues identified by resolving them as much as possible within the confines of the Canvas pre-existing framework. These included:

- applying commonly used Home and Section menu icons in all relevant places throughout the system, and reinforced linkages accordingly within and across modules;
- unifying the language use across the system and ensuring that all guidance notes targeting the end-users communicates directly to users (i.e. you) as to reinforce engagement;
- using plain icons with hover-over colour changes to increase the responsiveness of the system;
- organising all the discussion links within the two forums with six topics in each forum, and eliminating other discussion points linked to wellbeing-promotion exercises due to low usage;
- adding a ‘Support’ section in the main menu of the Home page where participants could raise a request for either technical or emotional support directly to the research team via emails; and
- for Canvas inbuilt issues that we could not change, we inserted additional guidance notes in COPe-support for users’ attention.

Lastly, we developed a navigation video and corresponding written guide to show participants the best way to navigate the system and use its various functionalities. We incorporated the technical know-how into user-friendly language with screenshots by pulling together all the feedback and suggestions from the study participants with the usage pattern data showing how the system was used in the real world.

## Discussion

Overall, the usability evaluation study provided comprehensive results to establish the usability and acceptability of the COPe-support prototype. The think-aloud test and remote usability test results demonstrated a high degree of usefulness, usability and acceptability as perceived by the target end-users (i.e. carers supporting a loved one affected by psychosis). The match of expectation and experience validated the significance of the iterative development process with active inputs from carers, service users, and professionals with relevant expertise in mapping out the essential ingredients and design of the intervention.^29,44–47^ Information on psychosis and related caregiving issues and the two interactive forums – one with experts, the other with carers as peers – were rated as relevant and helpful content. Participants identified that the anonymous online access and delivery of the intervention afforded them a high degree of flexibility and individuality. COPe-support had satisfactory utility in a real-world setting and its usability was supported by a heuristic evaluation using well-established usability benchmarking criteria.

This study, using a mix of online usability evaluation methods, helped establish the feasibility of providing an eHealth intervention for a critical mass of carers. The remote usability test was particularly helpful in establishing the accessibility, utility, ease of use and user-acceptance of COPe-support in the carers’ natural context. The qualitative feedback supplementing the quantitative evaluation results from both end-users and the eLearning expert also informed how best to refine the prototype in finalising COPe-support. Each method in isolation yielded useful insights around potential usability issues that the prototype might have, but no method on its own could identify issues from the perspective of both target users and usability experts while proposing potential solutions to those issues. By combining the findings from all three methods, this study demonstrates that we were able to obtain a holistic view of the usability of the system and identify positive changes to resolve those issues identified. Previous studies have similarly demonstrated the value of taking a mixed-methods approach to usability evaluation, with qualitative findings helping to support quantitative findings and generate solutions.^48,49^

The think-aloud test provided a means by which we could derive a comparatively in-depth understanding of how participants might interact with the intervention, and what key usability issues they might encounter. Since the number of participants was small, it was not expected that we would find all possible usability issues this way, but that this would identify any critical usability issues that might prevent users from engaging with the resource at all. This method allowed us to isolate where in the system key usability issues would occur, and to gather insights from participants about how they could be resolved in future development stages.

The remote usability test allowed us to gather a surface understanding of usability issues encountered by a wider group of potential users. It also gave a measure of general acceptance of the tool from a larger sample than the think-aloud test, making it a more reliable indicator of the overall suitability of the tool. However, while the remote test was able to identify general usability issues, it was not able to yield as much detail about the nature and specific causes of these issues, so could not inform the development of solutions to these challenges.

Unlike the think-aloud and remote usability tests, the heuristic evaluation provided a usability expert’s perspective on the intervention and was conducted within a framework of accepted best practice in user interface design. By virtue of comparing the COPe-support intervention with these accepted usability standards, the approach could identify both any shortcomings and reliable, widely tested solutions to resolve them. This is particularly crucial when evaluating prototypes, since by referring to established best practice guidelines the heuristic evaluation can anticipate potential usability issues that may not have been identifiable in the limited prototype, but which might emerge as the intervention grew to be fully-featured.

Our study recruitment and participation rate for the remote usability test adhered to the guidelines suggested by experts in the field and were satisfactory.^31,36,37^ The carers recruited through a range of NHS specialist mental health services across South East England were representative of the wider mental health carer population within the limitations created by the eligibility criteria. This should increase the generalisability of the study results. The remote usability test participation rate of 80% (*n* = 16 out of 20 consented) and its evaluation completion rate of 70% (*n* = 14) helped the study team to gauge a realistic participation and completion rate and to inform the sample size needed for a future effectiveness trial. These rates compare well with other similar studies investigating eHealth applications with general populations including carers.^50–53^ However, it is worth bearing in mind that whilst eHealth interventions seem advantageous in reaching target end-users, hence a relatively good recruitment rate in the beginning, completion rates of eHealth interventions are often lower than conventional intervention delivery via face-to-face formats.^8,49,54^ Paradoxically, the flexibility in access and usage that eHealth interventions offer to their users is arguably an unexpected contributing factor to users’ non-adherence or even incompletion.^8^ In the future clinical trial, in which the final version of COPe-support will be investigated for its clinical effectiveness in promoting carers’ wellbeing and other health outcomes, such inherent issues should be considered together with relevant implementation strategies to optimise retention and completion rates.^27^ The EFFIP project online intervention has the potential to provide psychoeducation, coupled with expert advice and peer support to carers, to fit their dynamic service needs and busy lifestyles given its online design and delivery medium. It also has the potential to be scaled-up and integrated with other online resources at a low cost.

The main limitation of this study is the use of a single eLearning expert rather than multiple experts in the heuristic evaluation. Due to human resource limitations, an alternative approach with multiple independent experts conducting the heuristic evaluation was not feasible. To mitigate possible bias and limits, we used a well-established heuristic evaluation tool to assess the 10 most important heuristics and relied on objective ratings of frequency and severity for the overall impact scores of each usability domain.^42,43^ Similarly, it is a limitation of the study that only three participants took part in the think-aloud study. It is widely acknowledged in the literature that the use of five subjects in such studies is needed to identify 80% of usability issues, although beyond this point adding more participants will be of limited effect in identifying further issues.^55,56^ However, in this instance the limitation was mitigated by the mixed-methods approach; although not all usability issues will have been identified from the think-aloud sessions alone, these were combined with insights from other methods. In addition, the remote usability test with a larger number of participants suggested a broad level of acceptance for the prototype, indicating that all urgent usability issues have been successfully identified. Another shortfall in the study design relates to the lack of feedback sought from the minority who did not take part in the remote test (*n* = 4, 20%) or from those who did not return the online evaluation (*n* = 2). Their disengagement might have been due to idiosyncratic usability and acceptability issues of COPe-support that some experienced but have not been covered by the comprehensive data collected thus far. An additional strategy to obtain subjective user experience data from those non- or incomplete-users, perhaps via a conventional medium (such as telephone or face-to-face), might help yield further insight.

## Conclusions

We developed an eHealth intervention providing information and psychosocial support for family carers supporting individuals affected by psychosis, called COPe-support. This usability study demonstrates that COPe-support was perceived as fit for purpose and had no notable usability issues that would pose a significant barrier to the end-users of the intervention. While the content of COPe-support was rated as helpful, relevant and useful, carers appreciated the flexibility and accessibility of a wealth of information and peer support afforded through the Internet-based design and delivery. The comprehensive data collected from potential end-users through the remote test in a naturalistic setting shed light on real-world usage pattern and usability issues. The insights from the expert-led heuristic evaluation and the think-aloud sessions with carers further informed the ways in which the identified usability issues could be best resolved. Such refinement work has since been undertaken to propel the prototype into the final draft of COPe-support. The next phase of the EFFIP project will see the intervention being tested for its effectiveness in promoting carers’ mental wellbeing and other health outcomes through an online randomised controlled trial.
